# Performance of the dentogingival junction with mta and biodentine on the treatment of invasive cervical resorptions. A literature review and case report

**DOI:** 10.4317/jced.57410

**Published:** 2021-01-01

**Authors:** Beatriz Gión-Guerra, Pablo Pérez-Lanza, Pedro Almiñana-Pastor, Pau Micó-Martínez, Francisco M. Alpiste-Illueca, Andrés López-Roldán

**Affiliations:** 1 Department of Stomatology, Faculty of Medicine and Dentistry, University of Valencia, Spain

## Abstract

Invasive cervical resorption (ICR) is an uncommon phenomenon (0.1%), however, it represents a challenge to the structural and functional integrity of the dentogingival junction, as well as a risk for the survival of the affected tooth. They are characterized by their location and invasive character, being able to appear in any tooth of the permanent dentition. It shows up after the damage to the cervical insertion apparatus, leaving the pulp without participation in the origin of the lesion. They may appear just below the junctional epithelium or at a more apical level. The MTA® (Dentsply, Tulsa dental, Tulsa OK) and the Biodentine® (Septodent, Saint Maur of Fossés, France) are two biomaterials that have demonstrated the ability to promote the neoformation of cement so they are considered an alternative in the treatment of the ICR. This article presents an ICR clinical cases treated with these biomaterials, in which favorable post-operative healing is observed.

** Key words:**MTA, Biodentine, biomaterials, root resorption, invasive cervical resorption.

## Introduction

Root resorption (RR) is defined as the loss of tooth hard tissue (dentin and cement), through the action of osteoclasts (1). These clastic cells are formed when mononuclear precursors derived from the monocyte-macrophage cell lineage are attracted to mineralized surfaces, fusing and adhering, increasing their reabsorptive activity (2). These functional clastic cells are responsible for the degradation of the calcified extracellular matrix, creating Howship´s gaps (1).

The pathway RANK (associated receptor with nuclear factor Kappa Beta) / RANK-L (the respective ligand) / OPG (Osteoprotegerin), is related to the osteoclasts activation. RANK-L, attached to another ligand protein (M-CSF), promotes the formation and activation of clastic cells which leads to increased bone resorption. This process, closely related to bone remodeling processes, is very similar to the ones that occurred in the RR. The periodontal ligament (PL) cells, pulp cells, odontoclasts and cementoclasts present OPG, which inhibits the clastic action of RANK-L (3). Without a resorptive stimulus, the PL cells express OPG, while the level of RANK-L increases during reabsorption. In the physiological processes of RR, as in the replacement of temporary teeth, the favorable RANK-L / OPG ratio seems to promote RR. Therefore, the break down of the equilibrium in this axis seems to be a trigger for the RR.

When speaking of RR in a pathological context, there must be a stimulus, which origin could be pulpal or bacterial. This could trigger a benign fibrovascular proliferation or a fibro-osseous disorder (4). The absence of epithelial remnants of Malassez (ERM) seems to be also linked. It seems that an injury or compromise of ERMs could be related to reabsorption, although more studies are needed to confirm it. Therefore, they can be considered a pathological process, with consequences for the survival of the tooth if not treated (1).

Linskog (2006) makes a classification of root resorptions, dividing them into 3 main types: by trauma, infection or hyperplastic, with the ICR in the last one.

In this paper we focus on cervical invasive resorption, so we will briefly review the characteristics. Hyperplastic reabsorption - the group to which the ICR belongs (5) - has an aggressive progression with a hard tissues inflammatory invasion. A differentiating factor of this third group is that simple elimination of the cause of the lesion is not effective in stopping the pathology. Removal of the resorptive tissue is essential to avoid recurrence, which could be due to residual infiltration in dentin tubules created by the inflammatory invasive tissue, which can connect to the PL in positions more apical to the initial defect (6).

ICR is a rare form of external RR with a prevalence of 0.1% (6). They are characterized by their cervical location and invasive character, being able to appear in any tooth of the permanent dentition. It appears after the damage to the cervical insertion apparatus, leaving the pulp without participation in the origin of the lesion. They may appear just below the junctional epithelium or at a more apical level (7).

In a clinical view, it is sometimes characterized by the presence of a “pink tooth” or “Mummery tooth“ (element for differential diagnosis with internal root resorption) as the highly invasive tissue becomes visible because of its high vascularity. If there is no external clinical signs, the diagnosis will be made by X-rays. Patients usually do not refer discomfort unless a secondary infections with pulpal or periodontal symptoms are present. The cause of this condition is uncertain, although predisposing factors such as trauma, internal bleaching or orthodontic treatment have been identified. These can alter or damage the cement bringing on the appearance of the process.

The treatment can vary depending on the progression degree of the disease, although for a correct treatment the entire inflammatory tissue must be removed and the restoration of the defect must be achieved in order to allow the attachment of the dento-gingival complex. ICRs are divided into 4 categories (6) depending on the involvement degree. In classes 1 and 2, pulp is protected and tissue has a fibrovascular structure. This level of affectation will determine the treatment and the success percentage, considering level 3 as a bad prognosis and 4 as untreaTable (case monitoring and subsequent extraction is considered as an option), due to the infiltrative nature of these lesions. Several techniques for the treatment of ICR are described from guided tissue regeneration to orthodontic extrusion. All of them with the same approach, the total elimination of the reabsorptive inflammatory tissue and reconstitution of the defect, aside from eliminating the stimulus if it persists (1). The following treatment depends on the progression of the resorption.

Precisely its location causes a situation of risk for the bacterial and immunological homeostasis of the oral cavity, since there is a destabilization in the nexus between the oral and internal environment. Although the active defense capacity of the epithelium and its cells is known, by the synthesis of defensins, calprotectin and lysosomes, the absence of a physical seal by the epithelium of attachment to the tooth supposes a situation of periodontal risk (8). As indicated, the elimination of the invasive tissue and the subsequent recovery of the defect is essential with the intention of recovering the integrity of the epithelial junction and regeneration of the periodontium. For regeneration and periodontal stability, a material that stimulates the formation of new cement is sought.

The MTA® was approved for use in people in 1998, for treatment of root perforations. Soon a clinical and biological response of the tissues appropriate for use in other areas was observed (9). This conglomerate of Portland cement, bismuth oxide, tricalcium silicate, tricalcium aluminum and silicate oxide does not irritate tissues and induces the regeneration of cement and PL (10). It seems that the biocompatibility observed can be attributed to the release of hydroxyl ions, with the increase in pH that it entails and the formation of calcium hydroxide during the process of hydration of the material (11). Its osteoinductive and cementogenic nature stimulates the release of lymphokines which favors remineralization . It has been observed that osteoblasts are able to adhere to the surface of MTA®, in the same way that a stimulation of PL fibroblasts occurs. A series of *in vitro* studies (12,13) indicate that the MTA® is a bioactive material, especially relevant in terms of cementogenesis and osteogenesis. A 2013 review (10) tries to draw conclusions, despite the diversity of methodology of the articles reviewed. All of them highlight the fact that new cement is formed on the MTA® and that, even in some cases, PL fibers are inserted onto the cement. Several inflammation degrees are observed, with different responses in some cases, which are always better in cases without bacterial contamination. In addition, due to its alkaline nature (pH 12.5), it is a bacteriostatic and bactericidal material, inhibiting the growth especially of the E. Faecalis.

As complications, the occasional presence of a fibrous capsule, as well as RR and ankylosis is highlighted. On the other hand, its setting time is slow (4 hours), with a curing period of up to 21 days, compromising its handling difficulty. Another material used in the treatment of reabsorbed processes is the Biodentine®. This is a calcium silicate cement, with a much shorter setting time (12 minutes) being the main advantage respect to the MTA®. One study observed that fibroblasts proliferate in the presence of Biodentine® (14) and that this presents similar levels of IL1-alpha and IL6.

Following is a series of cases with ICR treated with these materials and their subsequent evolution.

## Case Report

**Figure 1:**
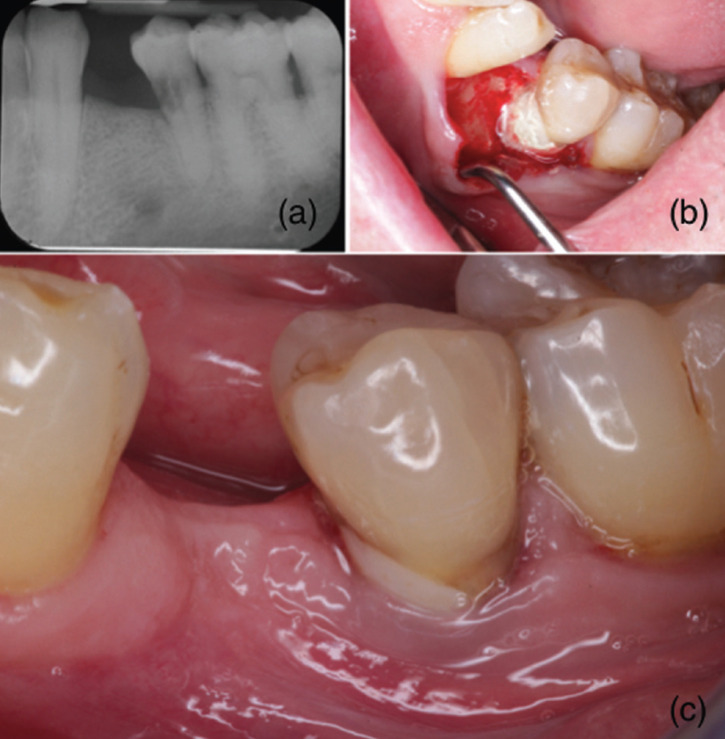
Radiograph of the external resorption in the coronal third of the root of tooth 3.5 (a); the surgical view after the treatment with MTA (b); clinical photography after three years (c).

Case 1: 50-year-old patient with no medical history of interest who, after radiographic and clinical examination, was detected an external resorption in the coronal third of the root of tooth 3.5, classified as grade II (6). Initially, the endodontics was performed with filling with lateral condensation technique, to later fill with Glass Ionomer (IV). 6 years later, in 2014, access surgery was performed with the aim of eliminating the old IV and replacing it with MTA®, since it had probing depth greater than 5mm with bleeding on probing. A surgical access was made with a mucoperiosteal flap. The previous obturation was eliminated and decontaminated with CHX. A basal layer of IV plus the surface layer of the MTA® was placed. Amoxicillin 500mg was prescribed for 7 days. One week later, a favorable healing was observed, and the non-absorbable polypropylene suture was removed. 3 years later, the tooth remains functional and the periodontal parameters were compatible with health (Fig. 1).

**Figure 2:**
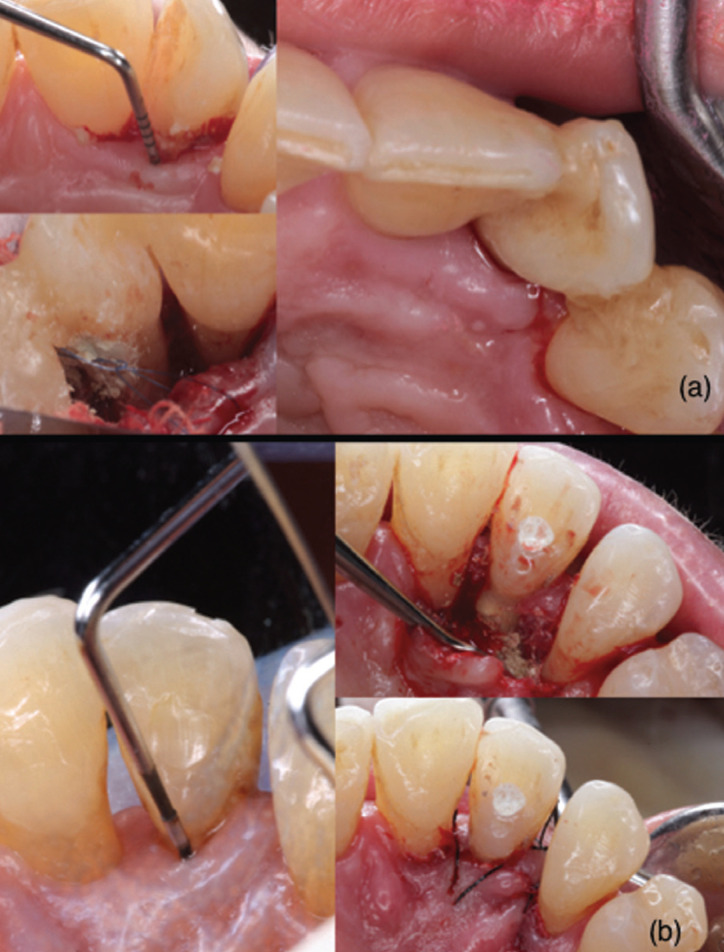
ICR on tooth 2.2 (a) with deep probing depth (b). The surgical view after the treatment with MTA (c).

Case 2: 61-year-old patient with no medical history of interest who was diagnosed with ICR on tooth 2.2. An sulcular incision was made, exposing and debriding the tooth and the root, and then the MTA® was placed. In this case, due to the extension of the lesion that was determined as Grade II (6) no root canal treatment was performed. The flap was sutured. The patient did not present discomfort to the postoperative period, although dentine hypersensitivity (DHS) appeared two years after, when root canal treatment was performed (Fig. 2a).

Case 3: 62-year-old patient, without medical history of interest. ICR was diagnosed in the palatine of tooth 1.2. A palatine approach was made with a mucoperiosteal flap for the exposure of the lesion and its curettage. It was filled with Biodentine® and sutured. Endodontic treatment was performed due to the degree of involvement of the piece, Grade II (6). 3 years later, the patient was sTable, with no signs of inflammation (Fig. 2b).

**Figure 3:**
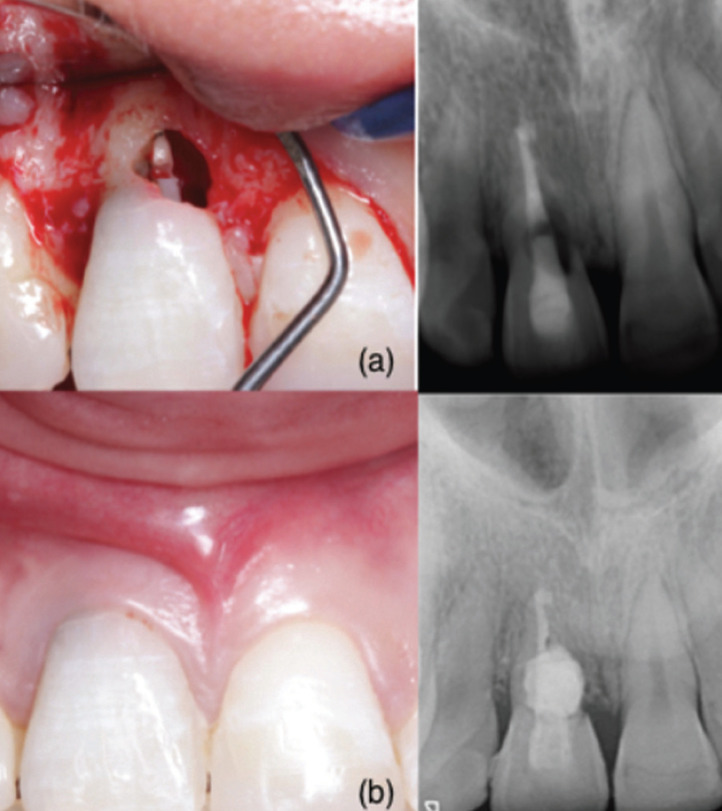
ICR in palatine of tooth 1.2 treated with Biodentine® and sutured (a); another patient with ICR in 1.1 treated with MTA and the evolution after six months (b).

Case 4: 14-year-old patient, with no medical history of interest, who was diagnosed with an ICR on tooth 1.1, with previous endodontic treatment due to post-traumatic necrosis. An unfavorable evolution was observed at the age of 18. At that time, it was diagnosed as Grade III (6). Intrasulcular incision was made preserving papillae, raising full thickness flap. Once debrided, the MTA® was placed. Antibiotics were prescribed, observing a favorable healing from the first week. 6 months later, the tooth remained asymptomatic (Fig. 3).

## Discussion

This paper presents the evolution of 4 cases that present ICR, with grades II and III (6). After their treatment, repetitive patterns were observed in all of them: absence of inflammation, probing depth compatible with health, absence of bleeding and pain. In one of them, which no conduit treatment had been performed prior to the intervention, DHS appeared. Therefore, it can be considered that after one year from the treatment the evolution is positive. It should be taken into account that if this intervention is not performed, the prognosis of these teeth would be unfavorable or impossible, so we delay the need for replenish that possible lost piece. This is of special relevance in young patients, in whom the placement of an implant is considered inadequate because of the bone development has not been completely finished, with the consequent vertical changes in the maxilla that will occur, which may lead to discrepancies in the gingival margins (15). The decrease in probing depth could be related to the regenerative capacity of cement and subsequent insertion of connective fibers (10), thus decreasing the risk of bacterial superinfection. However, histological studies would be necessary to support this hypothesis. One limitation observed for aesthetic restoration with MTA® is the gray translucency, which was not avoided despite the fact that the MTA® chosen was the white MTA® (wMTA®). In patients with a medium or high smile line it may be unsatisfactory for them, to which must be added the difficulty of handling the material.

Longer observation and evaluation periods are needed, in order to assess the long-term stability of these solutions. Nevertheless, the high biocompatibility of this material seems to offer good perspectives. It would be interesting to deepen the investigation of the periodontal tissues response, more specifically the dentogingival junction in contact with these biomaterials.
